# Crystal structure of 3-(tri­phenyl­phosphoranyl­idene)-2,5-di­hydro­furan-2,5-dione tetra­hydro­furan monosolvate

**DOI:** 10.1107/S2056989018011775

**Published:** 2018-08-24

**Authors:** Almaz Zagidullin, Daut Islamov, Elena Oshchepkova, Peter Lonnecke, Vasili Miluykov

**Affiliations:** a Arbuzov Institute of Organic and Physical Chemistry, FRC Kazan Scientific Center of RAS, Arbuzov Str. 8, 420088 Kazan, Russian Federation; b Department of Chemistry, Kazan State University, Kremlevskaya St. 18, 420008, Kazan, Russian Federation; c University of Leipzig, Faculty of Chemistry and Mineralogy, Institute of Inorganic Chemistry, Johannisallee 29, Leipzig, Germany

**Keywords:** crystal structure, tetra­hydro­furan solvate, pseudopolymorph, ylid

## Abstract

The title pseudo-polymorph of 3-(tri­phenyl­phospho­ranyl­idene)-2,5-di­hydro­furan-2,5-dione crystallizes with a tetra­hydro­furan solvent mol­ecule, *viz*. C_22_H_17_O_3_P·C_4_H_8_O. The succinic anhydride ring is approximately planar (r.m.s. deviation = 0.032 Å). The tetra­hydro­furan mol­ecule is disordered over two orientations about a pseudo-twofold axis with refined occupancy ratio 0.718 (4):0.282 (4). In the crystal, C—H⋯O hydrogen bonds link mol­ecules of the di­hydro­furan-2,5-dione derivative into chains parallel to the *b* axis and arranged into layers stacked along [100] alternating with hydrogen-bonded tetra­hydro­furan layers.

## Abstract 2

The structure of the pseudopolymorph of 3-(tri­phenyl­phospho­ranyl­idene)-2,5-di­hydro­furan-2,5-dione with a THF solvent mol­ecule is described. The compound has a hydrogen-bonded layer structure, and displays C—H⋯O hydrogen bonds connecting mol­ecules of the di­hydro­furan-2,5-dione derivative into chains.

## Chemical context   

Pseudopolymorphs are solvated forms of a compound that have different crystal structures and/or differ in the nature of the included solvent (Kumar *et al.*, 1999 ▸). The investigation of this phenomenon plays an important role for both fundamental and applied reasons. Phospho­rus ylides are useful inter­mediates, which have been used in many reactions and are involved in the synthesis of organic compounds (Selva *et al.*, 2014 ▸; Kolodiazhnyi, 1999 ▸; Balema *et al.*, 2002 ▸). In this paper, the structure of the pseudopolymorph of 3-(tri­phenyl­phospho­ranyl­idene)-2,5-di­hydro­furan-2,5-dione (Geoffroy *et al.*, 1993 ▸), crystallized with a THF solvent mol­ecule, is described.
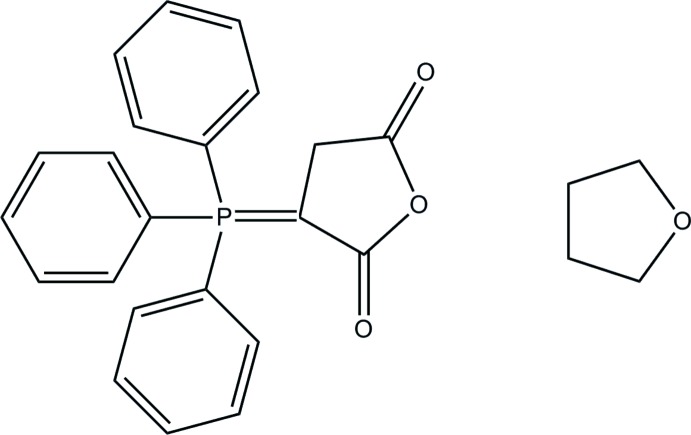


## Structural commentary   

**Figure 1:**
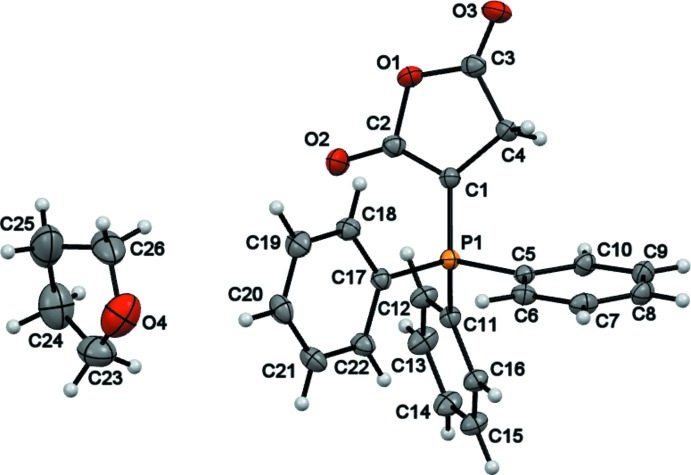
The mol­ecular structure of the title compound with displacement ellipsoids drawn at the 50% probability level. Only the major component of the disordered THF mol­ecule is shown.

In the title compound (Fig. 1 ▸), the succinic anhydride ring is almost planar (r.m.s. deviation = 0.032 Å), with the C4 methyl­enic carbon atom displaced by only 0.118 (2) Å out of the least-squares mean plane through atoms C1, C2, C3 and O1 [maximum deviation of 0.007 (2) Å for C2]. The phospho­rus atom deviates from the least-squares mean plane of the succinic anhydride ring by 0.1855 (4) Å. The arrangement of the phenyl rings is propeller-wise, which is common arrangement for Ph_3_P-*X* fragments. The THF solvent mol­ecule is disordered over two orientations related by a pseudo-twofold axis. As recently reported by Islamov *et al.* (2017 ▸), mol­ecules located in general positions rotate more easily than those located on symmetry elements, and the presence of disorder increases the number of minima on the profile of the rotational barrier, making the barrier even lower (Karlen *et al.*, 2010 ▸). However, since the quality of the anisotropic displace­ment parameters of the THF atoms is low, an attempt to determine the height of the rotational barrier using TLS analysis (Dunitz *et al.*, 1988 ▸) was unsuccessful.

## Supra­molecular features   

**Figure 2:**
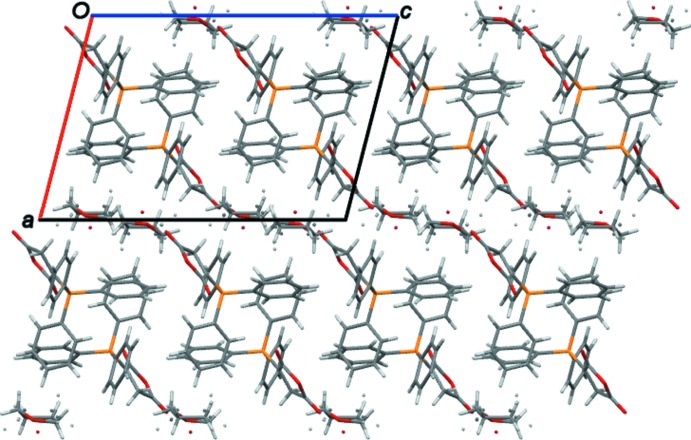
Crystal packing of the title compound viewed along the *b* axis.

In the crystal, 3-(tri­phenyl­phospho­ranyl­idene)-2,5-di­hydro­furan-2,5-dione mol­ecules inter­act through C—H⋯O hydrogen bonds (Table 1 ▸), forming chains running parallel to the *b* axis. Alternating layers of chains and THF mol­ecules are stacked parallel to the *bc* plane (Fig. 2 ▸) and connected by C—H⋯O hydrogen bonds (Table 1 ▸).

## Database survey   

**Figure 3:**
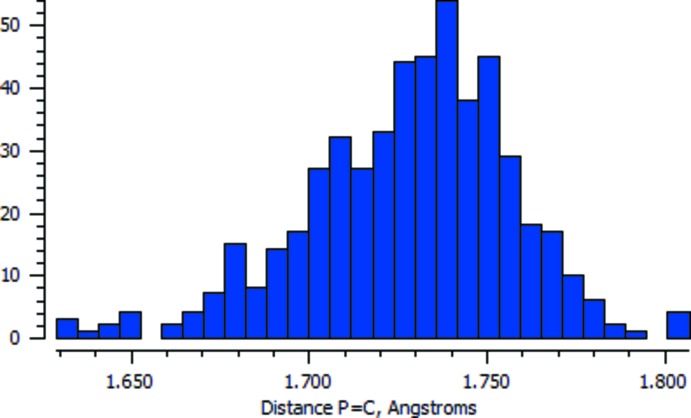
Distribution histogram of the P=C distance in Ph_3_P=C fragments.

**Figure 4:**
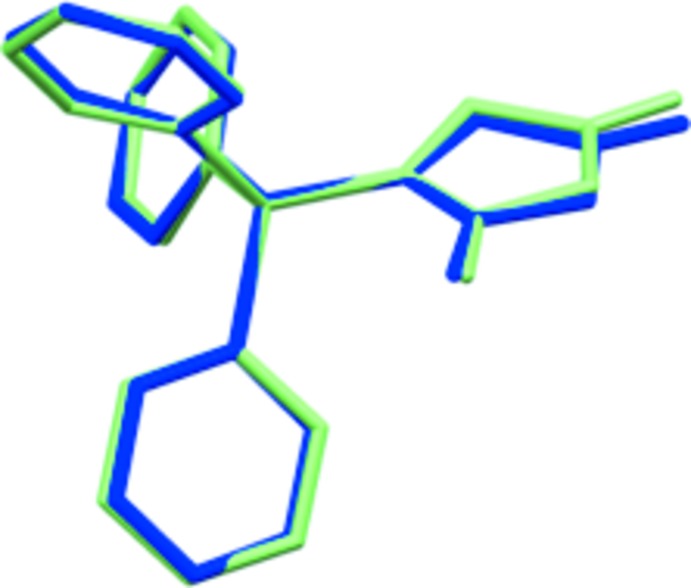
Structure overlay of THF solvate (green) and CH_2_Cl_2_ solvate (blue; Geoffroy *et al.*, 1993 ▸).

A search of the Cambridge Structural Database (Version 5.39, update February 2018; Groom *et al.*, 2016 ▸) revealed 426 structures containing the Ph_3_P=C fragment. The distribution histogram of the P=C distance [with a mean value of 1.729 Å and a standard deviation of 0.030 Å] is shown in Fig. 3 ▸. The P=C distance in the title compound is 1.717 (2) Å, which in good agreement with that of the di­chloro­methane pseudopolymorph [1.717 (6) Å; Geoffroy *et al.*, 1993 ▸]. In spite of the differences in the crystal packing, the conformation of the mol­ecule is very similar to that of the CH_2_Cl_2_ solvate (r.m.s. deviation = 0.032 Å; Fig. 4 ▸).

## Synthesis and crystallization   

To a stirred solution maleic anhydride (0.17 g, 1.72 mmol) in tetra­hydro­furan THF (5 mL) was added tri­phenyl­phosphine (0.45 g, 1.72 mmol) at room temperature. The reaction mixture was stirred at room temperature for 24 h, then the solution was filtered and concentrated under reduced pressure. The reaction mixture was allowed to cool in the freezer (243 K, three days) and yellowish crystals precipitated. The crystals were separated from solvent and dried to give 0.56 g (90%) of the title compound. ^1^H NMR (CDCl_3_, δ, ppm, *J*, Hz): 1.78 (*m*, CH_2_ from THF), 3.14 (s, 2H, CH_2_), 3.67 (*m*, OCH_2_ from THF), 7.44–7.72 (*m*, 15H, Ph). ^31^P{^1^H} NMR (CDCl_3_, δ, ppm, *J*, Hz): +13.6 (s).

## Refinement   

Crystal data, data collection and structure refinement details are summarized in Table 2 ▸. The THF mol­ecule is disordered over two sites with an occupancy ratio of 0.718 (4):0.282 (4). EADP and SAME restraints were used to model this disordered mol­ecule. The H atoms of the 3-(tri­phenyl­phosphor­anyl­idene) di­hydro­furan-2,5-dione mol­ecule were located in difference-Fourier maps and refined freely. The THF H atoms were placed geometrically and refined using a riding-model approximation with C—H = 0.99 Å, and with *U*_iso_(H) = 1.2*U*_eq_(C).

## Supplementary Material

Crystal structure: contains datablock(s) I. DOI: 10.1107/S2056989018011775/rz5234sup1.cif

Structure factors: contains datablock(s) I. DOI: 10.1107/S2056989018011775/rz5234Isup2.hkl

Supporting information file. DOI: 10.1107/S2056989018011775/rz5234Isup3.cdx

CCDC reference: 1862873

Additional supporting information:  crystallographic information; 3D view; checkCIF report

## Figures and Tables

**Table 1:** Hydrogen-bond geometry (Å, °)

*D*—H⋯*A*	*D*—H	H⋯*A*	*D*⋯*A*	*D*—H⋯*A*
C9—H9⋯O4*F* ^i^	0.92 (2)	2.59 (2)	3.253 (8)	129.4 (18)
C22—H22⋯O2^ii^	0.95 (2)	2.55 (2)	3.386 (2)	148.2 (16)
C20—H20⋯O4	1.00 (3)	2.58 (2)	3.452 (4)	145.8 (19)
C21—H21⋯O4*F* ^iii^	0.94 (2)	2.43 (2)	3.283 (6)	151 (2)

Symmetry codes: (i) 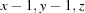; (ii) 
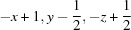
; (iii) 
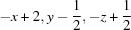
.

**Table 2:** Experimental details

Crystal data	
Chemical formula	C_22_H_17_O_3_P·C_4_H_8_O
*M*_r_	432.43
Crystal system, space group	Monoclinic, *P*2_1_/*c*
Temperature (K)	130
*a*, *b*, *c* (Å)	12.1287 (5), 10.5530 (4), 17.5838 (8)
β (°)	104.435 (4)
*V* (Å^3^)	2179.57 (16)
*Z*	4
Radiation type	Mo *K*α
μ (mm^−1^)	0.16
Crystal size (mm)	0.20 × 0.15 × 0.05

Data collection	
Diffractometer	Agilent Xcalibur Sapphire3 CCD
Absorption correction	Multi-scan (*CrysAlis PRO*; Agilent, 2014 ▸)
*T*_min_, *T*_max_	0.995, 1
No. of measured, independent and observed [*I* > 2σ(*I*)] reflections	30663, 7381, 5123
*R*_int_	0.061
(sin θ/λ)_max_ (Å^−1^)	0.758

Refinement	
*R*[*F* ^2^ > 2σ(*F* ^2^)], *wR*(*F* ^2^), *S*	0.062, 0.130, 1.03
No. of reflections	7381
No. of parameters	370
No. of restraints	10
H-atom treatment	H atoms treated by a mixture of independent and constrained refinement
Δρ_max_, Δρ_min_ (e Å^−3^)	0.55, −0.39

Computer programs: *CrysAlis PRO* (Agilent, 2014 ▸), *SHELXS97* (Sheldrick, 2015*a* ▸), *SHELXL2014* (Sheldrick, 2015*b* ▸), *Mercury* (Macrae *et al.*, 2008 ▸), *publCIF* (Westrip, 2010 ▸).

## supplementary crystallographic information

### Crystal data

**Table:**

C_22_H_17_O_3_P·C_4_H_8_O	*F*(000) = 912
*M**_r_* = 432.43	*D*_x_ = 1.318 Mg m^−^^3^
Monoclinic, *P*2_1_/*c*	Mo *K*α radiation, λ = 0.71073 Å
*a* = 12.1287 (5) Å	Cell parameters from 5863 reflections
*b* = 10.5530 (4) Å	θ = 3.0–32.6°
*c* = 17.5838 (8) Å	µ = 0.16 mm^−^^1^
β = 104.435 (4)°	*T* = 130 K
*V* = 2179.57 (16) Å^3^	Needles, pale yellow
*Z* = 4	0.20 × 0.15 × 0.05 mm

### Data collection

**Table:**

Agilent Xcalibur Sapphire3 CCD diffractometer	5123 reflections with *I* > 2σ(*I*)
Radiation source: sealed x-ray tube	*R*_int_ = 0.061
ω scans	θ_max_ = 32.6°, θ_min_ = 3.0°
Absorption correction: multi-scan (CrysAlis PRO; Agilent, 2014)	*h* = −18→17
*T*_min_ = 0.995, *T*_max_ = 1	*k* = −15→15
30663 measured reflections	*l* = −26→26
7381 independent reflections	

### Refinement

**Table:**

Refinement on *F*^2^	10 restraints
Least-squares matrix: full	Hydrogen site location: mixed
*R*[*F*^2^ > 2σ(*F*^2^)] = 0.062	H atoms treated by a mixture of independent and constrained refinement
*wR*(*F*^2^) = 0.130	*w* = 1/[σ^2^(*F*_o_^2^) + (0.0367*P*)^2^ + 1.6405*P*] where *P* = (*F*_o_^2^ + 2*F*_c_^2^)/3
*S* = 1.02	(Δ/σ)_max_ < 0.001
7381 reflections	Δρ_max_ = 0.55 e Å^−^^3^
370 parameters	Δρ_min_ = −0.39 e Å^−^^3^

### Special details

**Table:**

Geometry. All esds (except the esd in the dihedral angle between two l.s. planes) are estimated using the full covariance matrix. The cell esds are taken into account individually in the estimation of esds in distances, angles and torsion angles; correlations between esds in cell parameters are only used when they are defined by crystal symmetry. An approximate (isotropic) treatment of cell esds is used for estimating esds involving l.s. planes.

### Fractional atomic coordinates and isotropic or equivalent isotropic displacement parameters (Å^2^)

**Table:**

	*x*	*y*	*z*	*U*_iso_*/*U*_eq_	Occ. (<1)
P1	0.35862 (4)	0.49261 (4)	0.15957 (2)	0.01719 (10)	
O1	0.20198 (11)	0.76108 (12)	0.01566 (8)	0.0267 (3)	
O2	0.35896 (12)	0.79120 (13)	0.11583 (8)	0.0325 (3)	
O3	0.05937 (12)	0.67737 (15)	−0.07627 (8)	0.0365 (4)	
C1	0.26851 (14)	0.58651 (16)	0.09179 (10)	0.0196 (3)	
C2	0.28856 (15)	0.71485 (17)	0.08280 (10)	0.0228 (4)	
C3	0.13225 (15)	0.66384 (19)	−0.01654 (11)	0.0252 (4)	
C4	0.16138 (15)	0.54664 (18)	0.03290 (11)	0.0223 (3)	
C5	0.32090 (14)	0.33056 (16)	0.13491 (9)	0.0184 (3)	
C6	0.39680 (15)	0.24576 (18)	0.11459 (10)	0.0223 (4)	
C7	0.36203 (17)	0.12181 (18)	0.09439 (11)	0.0263 (4)	
C8	0.25270 (17)	0.08421 (18)	0.09336 (11)	0.0272 (4)	
C9	0.17691 (17)	0.16811 (18)	0.11337 (11)	0.0259 (4)	
C10	0.21095 (15)	0.29100 (17)	0.13488 (11)	0.0224 (3)	
C11	0.34766 (14)	0.51365 (17)	0.25923 (9)	0.0191 (3)	
C12	0.30953 (17)	0.62921 (19)	0.28046 (11)	0.0259 (4)	
C13	0.30127 (18)	0.6482 (2)	0.35722 (12)	0.0302 (4)	
C14	0.33144 (17)	0.5526 (2)	0.41208 (11)	0.0293 (4)	
C15	0.36981 (17)	0.4372 (2)	0.39104 (11)	0.0278 (4)	
C16	0.37760 (15)	0.41676 (18)	0.31450 (11)	0.0237 (4)	
C17	0.50533 (14)	0.51511 (16)	0.15815 (10)	0.0194 (3)	
C18	0.53141 (16)	0.56868 (18)	0.09223 (11)	0.0241 (4)	
C19	0.64382 (17)	0.5776 (2)	0.08833 (12)	0.0304 (4)	
C20	0.73049 (16)	0.5328 (2)	0.14956 (12)	0.0303 (4)	
C21	0.70505 (16)	0.4806 (2)	0.21537 (11)	0.0283 (4)	
C22	0.59299 (15)	0.47212 (19)	0.22015 (11)	0.0247 (4)	
H4A	0.1714 (16)	0.476 (2)	0.0002 (12)	0.023 (5)*	
H4B	0.0986 (18)	0.530 (2)	0.0566 (12)	0.028 (6)*	
H6	0.4736 (18)	0.273 (2)	0.1138 (12)	0.027 (5)*	
H7	0.4134 (16)	0.064 (2)	0.0801 (12)	0.021 (5)*	
H8	0.2294 (18)	0.001 (2)	0.0810 (12)	0.028 (6)*	
H9	0.1041 (19)	0.142 (2)	0.1131 (13)	0.033 (6)*	
H10	0.1598 (18)	0.349 (2)	0.1476 (13)	0.031 (6)*	
H12	0.2872 (18)	0.696 (2)	0.2434 (13)	0.033 (6)*	
H13	0.273 (2)	0.728 (2)	0.3708 (14)	0.044 (7)*	
H14	0.3238 (19)	0.566 (2)	0.4650 (14)	0.036 (6)*	
H15	0.3900 (19)	0.370 (2)	0.4293 (14)	0.035 (6)*	
H16	0.4058 (18)	0.332 (2)	0.2995 (13)	0.033 (6)*	
H18	0.4709 (18)	0.601 (2)	0.0507 (13)	0.032 (6)*	
H19	0.6601 (19)	0.614 (2)	0.0407 (14)	0.039 (6)*	
H20	0.811 (2)	0.539 (2)	0.1460 (14)	0.039 (6)*	
H21	0.7635 (19)	0.451 (2)	0.2572 (14)	0.037 (6)*	
O4	0.9797 (3)	0.6888 (3)	0.1470 (2)	0.0667 (10)	0.718 (4)
C23	0.9886 (4)	0.7506 (4)	0.2223 (2)	0.0567 (10)	0.718 (4)
H23A	0.9324	0.7145	0.2485	0.068*	0.718 (4)
H23B	1.0658	0.7385	0.2570	0.068*	0.718 (4)
C24	0.9665 (11)	0.8833 (6)	0.2065 (6)	0.0645 (14)	0.718 (4)
H24A	1.0263	0.9358	0.2408	0.077*	0.718 (4)
H24B	0.8917	0.9074	0.2153	0.077*	0.718 (4)
C25	0.9668 (12)	0.9006 (11)	0.1196 (4)	0.076 (2)	0.718 (4)
H25A	0.9144	0.9690	0.0946	0.092*	0.718 (4)
H25B	1.0442	0.9189	0.1136	0.092*	0.718 (4)
C26	0.9267 (7)	0.7765 (9)	0.0877 (3)	0.0600 (12)	0.718 (4)
H26A	0.8428	0.7709	0.0770	0.072*	0.718 (4)
H26B	0.9496	0.7601	0.0383	0.072*	0.718 (4)
O4F	1.0380 (5)	0.9389 (7)	0.1713 (4)	0.057 (2)	0.282 (4)
C23F	0.965 (3)	0.9030 (19)	0.2222 (17)	0.0645 (14)	0.282 (4)
H23C	0.8965	0.9582	0.2132	0.077*	0.282 (4)
H23D	1.0061	0.9077	0.2782	0.077*	0.282 (4)
C24F	0.9329 (11)	0.7719 (12)	0.1986 (6)	0.0567 (10)	0.282 (4)
H24C	0.8575	0.7504	0.2072	0.068*	0.282 (4)
H24D	0.9902	0.7114	0.2281	0.068*	0.282 (4)
C25F	0.930 (2)	0.771 (3)	0.1100 (8)	0.0600 (12)	0.282 (4)
H25C	0.9780	0.7024	0.0974	0.072*	0.282 (4)
H25D	0.8510	0.7605	0.0774	0.072*	0.282 (4)
C26F	0.975 (3)	0.894 (3)	0.0982 (12)	0.076 (2)	0.282 (4)
H26C	1.0239	0.8877	0.0612	0.092*	0.282 (4)
H26D	0.9116	0.9534	0.0759	0.092*	0.282 (4)
H22	0.5776 (17)	0.437 (2)	0.2658 (12)	0.026 (5)*	

### Atomic displacement parameters (Å^2^)

**Table:**

	*U*^11^	*U*^22^	*U*^33^	*U*^12^	*U*^13^	*U*^23^
P1	0.01787 (19)	0.0168 (2)	0.01680 (18)	0.00042 (16)	0.00412 (14)	−0.00023 (17)
O1	0.0279 (7)	0.0222 (7)	0.0280 (7)	0.0025 (5)	0.0034 (5)	0.0065 (5)
O2	0.0379 (8)	0.0195 (7)	0.0358 (8)	−0.0044 (6)	0.0013 (6)	−0.0017 (6)
O3	0.0282 (7)	0.0449 (9)	0.0310 (7)	0.0028 (6)	−0.0029 (6)	0.0112 (7)
C1	0.0203 (8)	0.0182 (8)	0.0191 (8)	0.0000 (6)	0.0027 (6)	0.0005 (6)
C2	0.0236 (8)	0.0212 (9)	0.0231 (8)	0.0020 (7)	0.0048 (7)	0.0006 (7)
C3	0.0203 (8)	0.0301 (10)	0.0254 (9)	0.0035 (7)	0.0061 (7)	0.0043 (8)
C4	0.0182 (8)	0.0238 (9)	0.0236 (8)	−0.0002 (7)	0.0024 (6)	0.0026 (7)
C5	0.0208 (8)	0.0170 (8)	0.0165 (7)	0.0008 (6)	0.0032 (6)	0.0013 (6)
C6	0.0224 (8)	0.0235 (9)	0.0204 (8)	0.0040 (7)	0.0041 (7)	0.0007 (7)
C7	0.0340 (10)	0.0213 (9)	0.0228 (9)	0.0082 (8)	0.0056 (7)	−0.0008 (7)
C8	0.0369 (10)	0.0159 (8)	0.0269 (9)	−0.0010 (8)	0.0043 (8)	0.0005 (7)
C9	0.0270 (9)	0.0205 (9)	0.0300 (9)	−0.0020 (7)	0.0066 (7)	0.0012 (7)
C10	0.0237 (8)	0.0183 (8)	0.0257 (9)	0.0017 (7)	0.0069 (7)	0.0010 (7)
C11	0.0190 (7)	0.0204 (8)	0.0186 (7)	−0.0006 (6)	0.0060 (6)	−0.0012 (6)
C12	0.0340 (10)	0.0216 (9)	0.0236 (9)	0.0040 (8)	0.0097 (7)	0.0020 (7)
C13	0.0427 (11)	0.0242 (10)	0.0281 (9)	0.0053 (8)	0.0170 (8)	−0.0012 (8)
C14	0.0375 (11)	0.0318 (10)	0.0218 (9)	0.0002 (8)	0.0135 (8)	0.0002 (8)
C15	0.0346 (10)	0.0272 (10)	0.0228 (9)	0.0023 (8)	0.0095 (8)	0.0037 (8)
C16	0.0269 (9)	0.0234 (9)	0.0221 (8)	0.0013 (7)	0.0082 (7)	0.0012 (7)
C17	0.0195 (7)	0.0201 (8)	0.0185 (7)	−0.0013 (6)	0.0047 (6)	−0.0016 (6)
C18	0.0237 (8)	0.0275 (9)	0.0217 (8)	0.0009 (7)	0.0069 (7)	0.0009 (7)
C19	0.0286 (10)	0.0383 (12)	0.0273 (10)	−0.0018 (8)	0.0125 (8)	0.0016 (9)
C20	0.0208 (9)	0.0387 (12)	0.0322 (10)	−0.0040 (8)	0.0084 (7)	−0.0051 (9)
C21	0.0208 (8)	0.0373 (11)	0.0246 (9)	0.0006 (8)	0.0014 (7)	−0.0034 (8)
C22	0.0238 (8)	0.0293 (10)	0.0206 (8)	−0.0004 (7)	0.0048 (7)	−0.0001 (7)
O4	0.079 (2)	0.0495 (17)	0.083 (2)	0.0060 (15)	0.0406 (17)	−0.0007 (16)
C23	0.059 (3)	0.069 (3)	0.039 (2)	0.002 (2)	0.0074 (19)	0.0047 (19)
C24	0.061 (2)	0.058 (3)	0.079 (5)	−0.004 (3)	0.027 (3)	−0.022 (3)
C25	0.050 (3)	0.061 (3)	0.113 (6)	−0.007 (2)	0.012 (5)	0.018 (4)
C26	0.0586 (19)	0.092 (3)	0.029 (3)	−0.0256 (19)	0.011 (3)	−0.001 (3)
O4F	0.051 (4)	0.076 (5)	0.037 (3)	−0.041 (3)	−0.004 (3)	0.012 (3)
C23F	0.061 (2)	0.058 (3)	0.079 (5)	−0.004 (3)	0.027 (3)	−0.022 (3)
C24F	0.059 (3)	0.069 (3)	0.039 (2)	0.002 (2)	0.0074 (19)	0.0047 (19)
C25F	0.0586 (19)	0.092 (3)	0.029 (3)	−0.0256 (19)	0.011 (3)	−0.001 (3)
C26F	0.050 (3)	0.061 (3)	0.113 (6)	−0.007 (2)	0.012 (5)	0.018 (4)

### Geometric parameters (Å, º)

**Table:**

P1—C1	1.7168 (17)	C17—C22	1.395 (2)
P1—C5	1.7952 (18)	C18—C19	1.385 (3)
P1—C17	1.8016 (17)	C18—H18	0.96 (2)
P1—C11	1.8039 (17)	C19—C20	1.387 (3)
O1—C3	1.360 (2)	C19—H19	0.98 (2)
O1—C2	1.455 (2)	C20—C21	1.384 (3)
O2—C2	1.212 (2)	C20—H20	1.00 (2)
O3—C3	1.201 (2)	C21—C22	1.385 (3)
C1—C2	1.392 (2)	C21—H21	0.94 (2)
C1—C4	1.506 (2)	C22—H22	0.95 (2)
C3—C4	1.502 (3)	O4—C26	1.423 (8)
C4—H4A	0.97 (2)	O4—C23	1.455 (5)
C4—H4B	0.97 (2)	C23—C24	1.440 (9)
C5—C6	1.393 (2)	C23—H23A	0.9900
C5—C10	1.397 (2)	C23—H23B	0.9900
C6—C7	1.393 (3)	C24—C25	1.540 (10)
C6—H6	0.98 (2)	C24—H24A	0.9900
C7—C8	1.380 (3)	C24—H24B	0.9900
C7—H7	0.95 (2)	C25—C26	1.460 (7)
C8—C9	1.383 (3)	C25—H25A	0.9900
C8—H8	0.94 (2)	C25—H25B	0.9900
C9—C10	1.385 (3)	C26—H26A	0.9900
C9—H9	0.93 (2)	C26—H26B	0.9900
C10—H10	0.94 (2)	O4F—C26F	1.404 (19)
C11—C12	1.388 (3)	O4F—C23F	1.461 (17)
C11—C16	1.395 (2)	C23F—C24F	1.468 (15)
C12—C13	1.393 (3)	C23F—H23C	0.9900
C12—H12	0.95 (2)	C23F—H23D	0.9900
C13—C14	1.381 (3)	C24F—C25F	1.549 (14)
C13—H13	0.96 (3)	C24F—H24C	0.9900
C14—C15	1.386 (3)	C24F—H24D	0.9900
C14—H14	0.97 (2)	C25F—C26F	1.445 (15)
C15—C16	1.389 (3)	C25F—H25C	0.9900
C15—H15	0.97 (2)	C25F—H25D	0.9900
C16—H16	1.02 (2)	C26F—H26C	0.9900
C17—C18	1.395 (2)	C26F—H26D	0.9900
			
C1—P1—C5	107.60 (8)	C17—C18—H18	119.1 (13)
C1—P1—C17	111.98 (8)	C18—C19—C20	120.22 (18)
C5—P1—C17	108.38 (8)	C18—C19—H19	118.5 (13)
C1—P1—C11	114.52 (8)	C20—C19—H19	121.3 (13)
C5—P1—C11	106.01 (8)	C21—C20—C19	120.07 (18)
C17—P1—C11	108.03 (8)	C21—C20—H20	120.2 (14)
C3—O1—C2	109.44 (14)	C19—C20—H20	119.7 (14)
C2—C1—C4	109.82 (15)	C20—C21—C22	120.18 (18)
C2—C1—P1	122.78 (13)	C20—C21—H21	120.4 (14)
C4—C1—P1	127.38 (13)	C22—C21—H21	119.4 (14)
O2—C2—C1	135.62 (17)	C21—C22—C17	119.98 (17)
O2—C2—O1	116.47 (16)	C21—C22—H22	118.8 (12)
C1—C2—O1	107.90 (15)	C17—C22—H22	121.3 (12)
O3—C3—O1	121.34 (18)	C26—O4—C23	107.1 (4)
O3—C3—C4	128.29 (19)	C24—C23—O4	107.0 (5)
O1—C3—C4	110.37 (15)	C24—C23—H23A	110.3
C3—C4—C1	101.89 (15)	O4—C23—H23A	110.3
C3—C4—H4A	109.7 (12)	C24—C23—H23B	110.3
C1—C4—H4A	114.2 (12)	O4—C23—H23B	110.3
C3—C4—H4B	107.5 (13)	H23A—C23—H23B	108.6
C1—C4—H4B	113.0 (12)	C23—C24—C25	105.1 (6)
H4A—C4—H4B	110.0 (17)	C23—C24—H24A	110.7
C6—C5—C10	119.98 (16)	C25—C24—H24A	110.7
C6—C5—P1	121.94 (13)	C23—C24—H24B	110.7
C10—C5—P1	118.06 (13)	C25—C24—H24B	110.7
C5—C6—C7	119.43 (17)	H24A—C24—H24B	108.8
C5—C6—H6	120.6 (13)	C26—C25—C24	101.1 (6)
C7—C6—H6	120.0 (13)	C26—C25—H25A	111.6
C8—C7—C6	120.15 (18)	C24—C25—H25A	111.6
C8—C7—H7	120.1 (12)	C26—C25—H25B	111.6
C6—C7—H7	119.7 (12)	C24—C25—H25B	111.6
C7—C8—C9	120.65 (18)	H25A—C25—H25B	109.4
C7—C8—H8	120.5 (13)	O4—C26—C25	104.8 (7)
C9—C8—H8	118.8 (13)	O4—C26—H26A	110.8
C8—C9—C10	119.81 (18)	C25—C26—H26A	110.8
C8—C9—H9	120.0 (14)	O4—C26—H26B	110.8
C10—C9—H9	120.2 (14)	C25—C26—H26B	110.8
C9—C10—C5	119.96 (17)	H26A—C26—H26B	108.9
C9—C10—H10	120.4 (13)	C26F—O4F—C23F	101.3 (16)
C5—C10—H10	119.6 (13)	O4F—C23F—C24F	103.2 (12)
C12—C11—C16	120.19 (16)	O4F—C23F—H23C	111.1
C12—C11—P1	118.66 (14)	C24F—C23F—H23C	111.1
C16—C11—P1	121.14 (13)	O4F—C23F—H23D	111.1
C11—C12—C13	119.78 (18)	C24F—C23F—H23D	111.1
C11—C12—H12	121.6 (14)	H23C—C23F—H23D	109.1
C13—C12—H12	118.6 (14)	C23F—C24F—C25F	103.2 (14)
C14—C13—C12	120.07 (18)	C23F—C24F—H24C	111.1
C14—C13—H13	121.2 (15)	C25F—C24F—H24C	111.1
C12—C13—H13	118.7 (15)	C23F—C24F—H24D	111.1
C13—C14—C15	120.23 (18)	C25F—C24F—H24D	111.1
C13—C14—H14	119.8 (14)	H24C—C24F—H24D	109.1
C15—C14—H14	120.0 (14)	C26F—C25F—C24F	102.9 (14)
C14—C15—C16	120.24 (18)	C26F—C25F—H25C	111.2
C14—C15—H15	120.3 (14)	C24F—C25F—H25C	111.2
C16—C15—H15	119.5 (14)	C26F—C25F—H25D	111.2
C15—C16—C11	119.48 (17)	C24F—C25F—H25D	111.2
C15—C16—H16	119.8 (13)	H25C—C25F—H25D	109.1
C11—C16—H16	120.7 (13)	O4F—C26F—C25F	108.1 (17)
C18—C17—C22	119.64 (16)	O4F—C26F—H26C	110.1
C18—C17—P1	119.62 (13)	C25F—C26F—H26C	110.1
C22—C17—P1	120.61 (13)	O4F—C26F—H26D	110.1
C19—C18—C17	119.90 (17)	C25F—C26F—H26D	110.1
C19—C18—H18	121.0 (13)	H26C—C26F—H26D	108.4

### Hydrogen-bond geometry (Å, º)

**Table:**

*D*—H···*A*	*D*—H	H···*A*	*D*···*A*	*D*—H···*A*
C9—H9···O4*F*^i^	0.92 (2)	2.59 (2)	3.253 (8)	129.4 (18)
C22—H22···O2^ii^	0.95 (2)	2.55 (2)	3.386 (2)	148.2 (16)
C20—H20···O4	1.00 (3)	2.58 (2)	3.452 (4)	145.8 (19)
C21—H21···O4*F*^iii^	0.94 (2)	2.43 (2)	3.283 (6)	151 (2)

Symmetry codes: (i) *x*−1, *y*−1, *z*; (ii) −*x*+1, *y*−1/2, −*z*+1/2; (iii) −*x*+2, *y*−1/2, −*z*+1/2.
